# The Role of Debt in the Maintenance of Homelessness

**DOI:** 10.3389/fpubh.2021.810064

**Published:** 2022-01-27

**Authors:** Nick Maguire

**Affiliations:** Centre for Innovations in Mental Health, Department of Psychology, University of Southampton, Southampton, United Kingdom

**Keywords:** debt, homelessness, organisational, individual, motivation, drug use, rent

## Abstract

Debt is highly prevalent within the homeless population. This narrative review makes use of a varied literature to explore the relationship between debt and homelessness, focussing particularly on organisational (systemic) and individual factors and formulating the relationship between the two. A number of forms of debt and the causes of that debt are explored, in terms of individual factors such as motivation and organisational factors such as inflexible rent protocols. These factors are considered within the context of the cash economy operated by people who are homeless, together with the survival behaviours that drive such a way of operating. Conclusions are drawn about the nature of interventions and how individual psychological approaches to motivation need to be taken into account.

## Introduction

Homelessness remains one of the significant indicators of damaging wealth differential in developed economies. In the UK in August 2020, 2,688 people were found to be sleeping on the streets of England (1). This was a drop from the previous year, the reduction being attributed to the “everyone in” initiative designed to reduce the incidence rate of COVID-19. This is however likely to be a significant underestimate of the total number of people who are homeless; Crisis (2) put the true figure of people who are homeless and living on the street at over 200,000.

There are many factors implicated in the causation and maintenance of homelessness (3), ranging from the way in which we configure our economy and geographical variation in wealth (4) right down to genetic predispositions of arousal (5). This multi-factored approach means that the causes and maintaining factors are highly complex in their interactions. Mental health factors, mainly associated with childhood abuse and neglect (6) are associated with behaviours that lead to tenancy breakdown, moderated by factors such as the environment (built and organisational), interpersonal relationships and economic factors. Those systemic factors can in turn be influenced by commissioning processes and government policy, which may be in turn led by economic and ideological processes.

The role of debt in the causation and maintenance, therefore, can be seen to be an interaction between individual behaviours (spending) and environmental factors (e.g. organisational and tenancy systems around rent payment, referral for court proceedings for non-payment, local authority support for rent arrears, etc.). In one of the few studies looking at debt-related mechanisms of homelessness in the Netherlands (7), loss of job and chronic shortage of income were cited as the most prevalent reason for debt (49% of respondents). Fines, credit buying (legal) and health costs (23%), use of drugs (18%) and gambling (10%) were also cited.

A number of reviews in both the peer-reviewed and grey literatures have covered financial difficulties generally across populations of people who are multiply excluded, but the research in this area is sparse. Attention in the literature tends to be paid to structural issues, such as mechanisms of financial exclusion [e.g., (8, 9)].

There is a particular dearth investigating the role of debt in the maintenance of homelessness for single homeless people. Given the lack of research, it is not possible to conduct a good quality systematic review. Rather a narrative review will be conducted on the available evidence. In addition, observations on systemic processes will be included, based on the author's experience. Given the existing literature on structural financial exclusion, this review will focus more on individual and organisational systemic factors that cause and maintain debt, which in turn contribute to a state of homelessness. Interacting roles of debt and how they interlink with other factors will be described. It will consider possible mechanisms whereby debt is implicated in the maintenance of homelessness and go on to develop ideas based on good practise, around interventions that may reduce the impact of this relationship.

## Structure of Paper

This paper is divided into four main sections. The methodology describes the search strategy and papers included. The main section details a number of forms of debt experienced by people who are homeless, some related to each other, some separate. A section on a framework describing cash economies considers this as a maintaining factor, before moving to the final main critical section on possible interventions and the lack of psychological thinking. A concluding section summarises the thinking that results from the evidence considered.

## Methodology

Several databases were searched with the simple search terms “homeless^*^ and debt”. The search strategy started broad with Google Scholar and Google (to pick up any charity and other stakeholder reports that were not identified as academic literature). In addition, a meta-search engine was used (that searches CINAHL, Medline and PsychInfo). A citation search was used, as well as the author's own information on relevant papers. No exclusion criteria were applied and no date range set. All papers identified were written in English.

A mix of qualitative and quantitative papers, together with narrative reviews were identified, together with several reports commissioned and produced by homelessness charities. The literature covers research carried out in the UK, Scandinavia, Europe and the US. Different health ecosystems are therefore represented in factors leading to debt. The search was not intended to be exhaustive, but rather provide evidence where it existed on mechanisms of debt in homelessness. See Table 1 for a summary of papers identified and used to identify the role of debt below.

**Table 1:** Papers on homelessness and debt identified through literature search.

**Author and year**	**Peer review?**	**Design**	**Participant Population and country**	**Construct under investigation**	**Results**	**Comments**
Amar et al. (10).	Yes	Experimental design involving debt repayment simulation	171 people recruited from a non-clinical population. US	Effective interventions for debt	Small debts tend to be paid off before larger ones, despite this being problematic in terms of interest.	Useful experimental design, underpinning thinking and behaviour that may be important in debt management generally.
Bernard and Casey, (11)	No	Qualitative methods, interviews	Front line workers and people with lived experience of homelessness. Number not stated. UK	Debt associated with UC and how it maintains homelessness	Several issues around UC causing debt identified. Recommendations made to change systems of administration.	UK report by homeless charities making use of qualitative and observational data to formulate UC issues in debt.
Culhane et al. (12)	Yes	Secondary data analysis. Tests of difference among multiple populations	4679 people who are homeless who experience severe mental health issues. US.	Effectiveness of supportive housing	Supportive housing is effective in enabling reductions in other services, e.g. hospitalisations, time incarcerated. Debt costs reduced.	Supportive housing may be an intervention that should be considered in dealing with debt. Housing first in the UK may serve this purpose.
Emmet (13)	No	Qualitative methods	19 people who were homeless or at risk of homelessness. UK	Mechanisms of housing debt and its effects.	Changes in circumstances and unexpected expenditure placed people at risk of housing debt. Housing debt placed significant emotional strain on people, particularly families.	Identifies the role of housing debt in difficulties in maintaining tenancies, meaning a vicious cycle is established. Stress may be a moderating factor to be investigated.
Flåto and Johannessen (14)	No	Qualitative methods and review of literature	Economic and social behaviours of people who are homeless. Norway.	‘Harvesting economy', i.e. the rational behaviours associated with managing cash and debt.	Economic behaviours result in short-termism and reliance on social relationships and networks to manage day to day life.	Really useful framework to consider rational behaviours that may underpin debt maintenance.
Mogk et al. (15)	Yes	Cross sectional survey	101 people who were homeless. US	Impact of legal structures and practise on homelessness.	Strong association between legal involvement and consequences such as fines, and homelessness status. Possible maintaining factors.	Useful to identify financial factors implicated in maintenance of homelessness.
O'toole et al. (16)	Yes	Cross sectional survey	274 people, around half of whom were homeless. US	Impact of accumulation of medical debt on health seeking behaviours	Aggressive debt recovery had a significant negative effect on the health seeking behaviours of people who were homeless.	The impact of debt on health as an important maintaining factor in health inequality is identified.
Skolnik (17)	Yes	Narrative review	NA	Role of punishment in maintenance of homelessness	Framework of alternative punishments generated that are hypothesised to reduce contribution to homelessness	Useful to consider in the context of Mogk et al. (2020) as alternative to standard methods of dealing with debt.
van Laere et al. (7)	Yes	Cross sectional survey. Self report historical and current data gathered at one time point.	120 people recently made homeless. Netherlands	Factors implicated in the causation and maintenance of homelessness.	A number of factors identified in pathway to homelessness, including debt for those evicted from tenancies.	Debt was significantly implicated in eviction for those who had lost tenancies. Provides context for the observations around systemic issues discussed.

## Forms of Debt in Homelessness

According to the literature covered and in the author's experience, people can be in financial debt in varying of forms, that have differential impacts on their lives. The main forms of debt to be covered in this review are: rent debt; drug debts; debts to peers; debts caused by benefits systems; debts caused by health treatment; and debts caused by court-imposed fines. The important issue is that most of these forms of debt are implicated for different reasons in repeat tenancy loss.

### Drug Use as a Setting Condition for Debt

People who are homeless, in the main, have significant and complex issues, involving functional interactions between mental health, drug and alcohol and social relationship issues (3). Good evidence suggests that these issues are strongly associated with early childhood abuse and neglect (18). This means that the behaviours that lead to tenancy breakdown can be considered to be associated with ways of coping with these adverse childhood experiences. For example, difficulties in regulating emotions have been found to be associated with childhood abuse [e.g., (19)], and in turn, drug and alcohol use have been found to be ways of self-medicating for artificially regulating emotions. Drug and alcohol use become habitual over time, serving several functions such as avoidance of uncomfortable withdrawal experiences. It can therefore be argued that from the individual's perspective, a financial priority is to continue to fund habitual drug or alcohol use, particularly if the person is multiply and chronically excluded from statutory health, social and housing services. Less debt is accrued through sole abuse of alcohol, as drink is much cheaper to acquire. Thus, a set of interacting factors can be seen to lead people who are homeless into behaving in ways that, as will be seen, mean they are more likely to accrue debt.

### Rent Debt

Emmet (13) summarised sources of significant housing debt for those who are homeless. Rent debt accrued through varying income was a significant factor, leading sometimes to physical ill-health as well as mental health and other emotional issues. Most housing solutions require regular payment of rent and in some cases service charges (e.g. homeless hostels, social housing, private rented sector). Non-payment of rent is one of the most common reasons for eviction, and therefore maintenance of homelessness. Further, debts accrued through non-payment of rent are often carried forward and used as reasons not to house people until those debts are repaid. Therefore, prioritisation of substances over rent can be seen to result in exclusion *via* two mechanisms; firstly, non-payment of rent, and secondly, debts accrued being carried forward. Many tenancies (including hostels) will not be available for people with rent debt.

This is mostly an issue for those who use illegal drugs, due to the significant daily cost compared to the relatively cheap alcohol use. The pattern of antisocial behaviours associated with alcohol and drugs are different; drug use is mainly associated with acquisitive criminal behaviours and debt accrued, whereas alcohol tends to result in behaviours of disinhibition, such as verbal and physical assault.

There are also systemic issues that increase the likelihood of rent debt. Many of the debts accumulated by people who are homeless can be seen to be a function of poorly configured organisational systems that do not take account of the individual needs of people who are homeless. For example, many forms of temporary accommodation (e.g., hostels and shelters) operate “service charges” that are additional charges levied automatically to pay for provision of meals and other services. If these forms of charge are implemented without flexibility or thought, people who do not use the services feel aggrieved about paying them. This sometimes leads to non-payment of those service charges, but as they're rolled into the rent, that doesn't get paid either. Thus the accumulation of rent debt results from an inflexible system in which charges that do not applied to all residents are automatically levied. Other systems charges such as deposits can also be barriers to obtaining tenancies.

### Peer and Dealer Debt

Another form of debt accrued can be to peers and dealers, particularly around illegal drug use. Drug dealers are in the business of dependency, and will very often “tick” users, i.e. supply them with payment due at a later date. Thus, the user can ramp up a significant debt over time, which has to be repaid to avoid sometimes violent consequences. The level of debt can be kept stable by the dealer, therefore ensuring that the user always must return, resulting in continued dependence. Thus, debt can be used as a way of ensuring continued income through sustained dependence.

Debts to peers have a range of consequences and are important in maintaining homelessness. People are less able to fund recovery activities if the priority is to repay peers who have lent them money, often to pay off another form of debt. Not only does this form of debt mean that practical forms of recovery cannot be funded, but this debt may be used as leverage for other risky behaviours that may lead to tenancy breakdown (e.g. dealing and stealing). Thus, debt is used by dealers in order to extract a range of behaviours from vulnerable people, including staying in debt through use, or other illegal behaviours that may lead to imprisonment.

Serious debt to drug dealers can result in physical harm and even death, if not being drawn into working for those dealers. This of course increases the risk of arrest and incarceration. Experience of recent drug supply patterns attributable to the COVID pandemic (e.g. “county lines” dealing networks) indicates that this issue is worsening.

### Court Debts

A significant form of debt implicated in the maintenance of homelessness is costs accrued through illegal activity that is considered by the courts. This is often in the form of fines that must be paid, often over time as part of an agreement. In a recent study, Mogk et al. (15) found that over 25% of their US sample reported unpaid fines levied by the courts. Importantly, individuals with this kind of debt reported 22.9 months more homeless time than those without, once demographic factors were controlled for. Skolnik (17) provides some useful ways of reconsidering punishments that do not exacerbate debt, including more proportional financial penalties such as day fines, and other fundamental interventions such as absolution frameworks. Thus, the dynamic relationship between the behaviours that lead to homelessness and the financial consequences of some of those behaviours can be seen to be played out through debt. The debts are often not repaid quickly and can act as a barrier to further tenancies.

### Benefits Debt

In the UK, universal credit (UC) has provided significant difficulties for many people who are homeless (11). A number of mechanisms underpin these issues. Firstly, new accounts required 5 weeks to set up, requiring individuals to sustain themselves for that period. Thus, individuals were likely to accrue debt over that period, from a range of people including local authorities, drug dealers, peers, and landlords. This was recognised and individuals were enabled to borrow money for the period of time until regular UC payments started. However, this is still a debt, to be paid back over a period of 12 months and would still result in a monthly deficit. Additional difficulties are around the 1-month arrears in which UC is paid, and the removal of systems to pay rent directly to landlords. For people who are homeless and have addiction issues, prioritisation of habit over rent payment is common. Systems that can ensure that rent is paid first can be part of a useful systemic intervention to overcome this prioritisation.

Any historical debts, accrued through loans and rent arrears are often paid back through involuntary attachments to UC, resulting in significant deductions. This can leave people who are homeless with very little to live on a week, increasing the chances of funding drug and alcohol through debt and antisocial behaviours.

Lump sums paid to people who are living on the streets or in insecure accommodation can be problematic, particularly if people have drug and alcohol problems. Many people describe having access to means (money) leading to increased use as long as the money was available. Benefits and other finance systems (e.g. loans for housing set up) are often set up to pay money in lump sums rather than over time. Thus, the systems set up individuals who have drug and alcohol problems to fail in terms of spending large amounts of money in short spaces of time. This can result in debt if the money is to be paid back.

### Health Debt

Health debt varies significantly by country and by income. In countries where there is no universal health system [e.g. the US, in which 28m people remain uninsured; (20)], debt is incurred through e.g. emergency room interventions or other essential procedures. Health companies are increasingly and more aggressively chasing such debt, resulting in people e.g. losing their homes through bankruptcy (16). This is particularly true of those already in poverty.

### Finance in Housing

Even when housed, a number of structural factors increase the likelihood of building up debt, or become unmanageable as a result of debt.

Culhane et al. (12) identified debt service costs (e.g. non-payment of rent and unoccupied space, benefit subsidy) in a US population as a significant cost that can be reduced in supported housing interventions. They do not, however, discuss how these arrears are incurred or the specific mechanisms whereby personal debt is translated to benefit debt that can be saved through the intervention described. However observations made in a number of private and hostel dwellings illustrate some of the mechanisms of accruing debt.

Non-payment of rent has already been discussed. But there are other more subtle ways in which debt is a product of and contributes to homelessness. For example, utilities are often metered, as a result of landlord experiences of tenants who did not pay bills. This experience is generalised to all those who are perceived as high risk, and together with lack of access to bank accounts to pay direct debits, means that payment metres are widely used. This also means higher tariffs and more expensive energy. In addition, the cash economy most often used by people who are homeless results in uneven distribution over time, resulting in weeks when no money is available. For some, this lack of money can be evened out by pawning possessions or borrowing money in order to pay for food and energy. For some, more extreme measures are needed at such times, such as stealing, begging or dealing drugs.

### Cash Economy

The cash economy that many people who are homeless use provides significant barriers for stable budgeting. As discussed, payment of benefits for many is in lump sums every four weeks. This means that issues other than payment of regular bills may take priority, thereby building up debt. Particularly common are drug and alcohol addiction issues. People will prioritise reducing withdrawal and other physiological and psychological factors over reduction in debt in other areas.

Flato and Johannessen (14), using a qualitative methodology, surmised that people who are homeless operate a “harvest” economy', which enables money to be gradually accumulated and quickly spent. They argue that this is an entirely rational way of operating financially, given that they are at high risk of being robbed, thus carrying around cash for any length of time makes little sense. Activities designed to gather money (stealing and begging) leave people vulnerable, so when enough has been “harvested” for a particular activity (e.g. buying drugs, food etc.), it is immediately spent. The participants reported paying off debts if they had money, but also incurring debts for others (e.g. buying drugs for them) with the expectation that the debt would be paid later. This could be a variable strategy.

## Interventions

Interventions can be broadly divided into three types. Most of the literature covers two; structural and systemic levels of change, i.e. change in the societal and financial structures that govern the way in which the country is run, and change at the level of the organisational systems with which the individual interacts. These two can in some cases interact, e.g. banking operates with levels of security and ID that may be considered structural, but also systemic in terms of the way in which local systems operate to the exclusion of the individual (e.g. bank decisions about direct payments to landlords). The third level of intervention is with individuals, e.g. budget training.

Structural interventions are covered in some detail elsewhere [e.g. (8)] so will not be covered here, although some novel frameworks do add to the existing literature. For example, Slonik (17) proposes a heuristic whereby quality of life ordinances are contravened by people who are homeless, that elicits various forms of punishment from society. As this plays out financially, they propose that the state should adopt a much more sophisticated set of parameters around payment of fines, including “debt absolution frameworks”, overhauling criminal and civil methods for dealing with fines that remain unpaid.

### Budgeting

Many interventions focus on teaching budgeting skills to people who are homeless, modelled on other interventions that have been found to be useful with general populations [e.g. (10)]. However they often do not deal with issues of motivation to make behavioural changes. The difficulties around addiction can be much more powerful than the consequences of debt, meaning that motivation to make use of new skills, even if effectively learned, can be low. Budgeting skills may be useful, but only when an individual is ready to make behavioural change, so implementation of this intervention needs to be intentionally related to the person's assessed motivation.

### Systemic Interventions

Organisations should look at the costs that they attach to tenancies of all forms. In particular, charges such as service charges that may be resented by tenants and not paid by UC should be examined (11), and flexibility in implementation may be warranted.

The way in which rent is collected should be re-examined, particularly in terms of direct payments to landlords. This system can be abused so does need to be implemented in partnership with the individual, through discussion around priorities.

Organisations who house people who are homeless should not be setting them up for failure by not attending to the known and predictable behaviours associated with build-up of debt. If someone coming into a tenancy has debt, a clear assessment is needed of the underlying reasons for that accrual. It may be that more purposive, informed decisions may be taken that make success more likely. Organisational factors such as assessments often do not take account of an individual's level of motivation to address these long-standing issues.

Existing interventions often involve providing loans (and sometimes gifts) to people to cover rent deposits and guarantee further debt accrual. These systems are often run by local authorities, but sometimes voluntary sector organisations such as local homelessness charities.

### Underpinning Psychosocial Mechanisms of Change

In order for individuals in change at any level, issues of motivation to engage in the process of change need to be worked through. A significant reason for the failure of such interventions is that these issues are not even considered. It is often assumed that the person who is homeless will automatically be intrinsically motivated to make those changes, as they make sense to most people. However, as Flåto and Johannessen (14) identify, from the person's perspective, the behaviours leading to debt are an entirely rational way of coping when living on the streets.

Psychological interventions such as Motivational Interviewing [e.g. (21)] or other interventions that work with the individual and where they are in what they want to change need to be used as possible methods of change at the individual level, to overcome such mechanisms. These forms of intervention should be “baked in” to the wider financial or organisational interventions rather than being seen to be “bolt on”. Only in this way will a multidimensional approach that takes account of the dynamic relationships between mental health, housing, behavioural, psychological and financial factors be understood in a way that is going to be useful to the person. This requires collaborative ways of working across organisations, something that is notoriously difficult to achieve in a sustainable way given different epistemological and practical beliefs and approaches.

## Conclusions

This review sought to understand the way in which debt is implicated in the maintenance of homelessness. Through consideration of the existing literature and observational evidence, a picture emerges of a dynamic set of relationships between multiple factors, including understandable, “rational” behaviours of people, and the often inflexible, mindless responses of systems and structures. In order to progress with this issue in any meaningful way, we need to understand the mechanism of accrual of debt in terms of the interaction of the individual and their environment, rather than focusing on either in isolation. Societal and structural ways of viewing and treating people who are homeless can be seen to be the setting conditions for unhelpful systemic attitudes, which then play out in mindless maintenance of debt through inflexible punishments and consequences. These punishments are assumed to act as deterrents, but as discussed if there is little motivation to change behaviours, the deterrent function is largely redundant and in fact makes the problem worse, and more debt likely.

Debt can therefore be seen to be one of the fundamental factors maintaining homelessness, overseen by crudely modelled financial systems. Interventions need to be not only with individuals, but the systems that operate those crude processes also, to knit together an understanding of why people behave in such a way that accrues debt, with the complicity of their surrounding systems. Interventions such as those provided by Amar et al. (10) for populations of people who are housed should be examined for relevance and adaptation to make them more effective for people in the homeless population.

When implementing interventions on the individual level, motivation to change has to be a consideration and engagement techniques built into any work done. Therefore, not only do we need to pay attention to systemic and individual factors, but also the motivation of individuals and those that set up and run those systems to engage in change.

Future research needs to unpack some of those dynamic relationships to understand individual attitudes to financial management, from the perspectives of both the people experiencing the debt and those who deal with the consequences. Evaluation of the unintended consequences of ways of configuring systems may illuminate some of the mechanisms of exclusion and exacerbation of debt.

This review is just the start of a process of identifying factors implicated in threat at a range of levels, as well as the interactions between those levels. It identifies the gaps in the literature around individual and systemic factors in debt, that may over time be addressed.

## Author Contributions

NM is the sole author of this work and bears sole responsibility for the content.

## Conflict of Interest

The author declares that the research was conducted in the absence of any commercial or financial relationships that could be construed as a potential conflict of interest.

## Publisher's Note

All claims expressed in this article are solely those of the authors and do not necessarily represent those of their affiliated organizations, or those of the publisher, the editors and the reviewers. Any product that may be evaluated in this article, or claim that may be made by its manufacturer, is not guaranteed or endorsed by the publisher.
